# A Buoy for Continuous Monitoring of Suspended Sediment Dynamics

**DOI:** 10.3390/s131013779

**Published:** 2013-10-14

**Authors:** Philip Mueller, Heiko Thoss, Lucas Kaempf, Andreas Güntner

**Affiliations:** 1 Helmholtz Centre Potsdam, German Research Centre for Geosciences, Section 5.4 Hydrology, Telegrafenberg, Potsdam 14473, Germany; E-Mails: Heiko.Thoss@gfz-potsdam.de (H.T.); Andreas.Guentner@gfz-potsdam.de (A.G.); 2 Helmholtz Centre Potsdam, German Research Centre for Geosciences, Section 5.2 Climate Dynamics and Landscape Evolution, Telegrafenberg, Potsdam 14473, Germany; E-Mail: lucask@gfz-potsdam.de

**Keywords:** buoy, monitoring, suspended sediments, lake, turbidity, connectivity

## Abstract

Knowledge of Suspended Sediments Dynamics (SSD) across spatial scales is relevant for several fields of hydrology, such as eco-hydrological processes, the operation of hydrotechnical facilities and research on varved lake sediments as geoarchives. Understanding the connectivity of sediment flux between source areas in a catchment and sink areas in lakes or reservoirs is of primary importance to these fields. Lacustrine sediments may serve as a valuable expansion of instrumental hydrological records for flood frequencies and magnitudes, but depositional processes and detrital layer formation in lakes are not yet fully understood. This study presents a novel buoy system designed to continuously measure suspended sediment concentration and relevant boundary conditions at a high spatial and temporal resolution in surface water bodies. The buoy sensors continuously record turbidity as an indirect measure of suspended sediment concentrations, water temperature and electrical conductivity at up to nine different water depths. Acoustic Doppler current meters and profilers measure current velocities along a vertical profile from the water surface to the lake bottom. Meteorological sensors capture the atmospheric boundary conditions as main drivers of lake dynamics. It is the high spatial resolution of multi-point turbidity measurements, the dual-sensor velocity measurements and the temporally synchronous recording of all sensors along the water column that sets the system apart from existing buoy systems. Buoy data collected during a 4-month field campaign in Lake Mondsee demonstrate the potential and effectiveness of the system in monitoring suspended sediment dynamics. Observations were related to stratification and mixing processes in the lake and increased turbidity close to a catchment outlet during flood events. The rugged buoy design assures continuous operation in terms of stability, energy management and sensor logging throughout the study period. We conclude that the buoy is a suitable tool for continuous monitoring of suspended sediment concentrations and general dynamics in fresh water bodies.

## 1. Introduction

Understanding suspended sediment dynamics (SSD) has been an important topic in both applied and process research for several decades. From different perspectives such as hydrology (e.g., [1]), hydraulic engineering (e.g., [2]) and sedimentology (e.g., [3]), scientists have investigated SSD in catchments and in the reservoirs, lakes and estuaries into which the watersheds drain.

Duvert *et al.* [4] pointed out the necessity of monitoring suspended sediments, especially in rural areas that are situated in small mountainous catchments, because these catchments are increasingly facing human pressures due to land use changes. Many recent hydrological studies, therefore, have focused on upland erosion, the resulting reservoir siltation and reduction of reservoir capacity [5,6]. Reservoir siltation can cause a serious reduction in water availability for a given region. Viseras *et al.* [7] showed that the storage capacity of a reservoir in Spain decreased by around 80% within a 20-year period. Besides reservoir siltation, erosion and sediment transport may also cause a loss of valuable farmland [8] and damage to hydro-electrical facilities [9].

To understand the driving forces and effects of SSD and to develop sustainable mitigation measures, it is necessary to understand the linkage between different landscape units in a catchment that act as source, conveyor and sink for sediments. This linkage is represented by the term “hydrological connectivity”. In a hydrological sense, Pringle [10] describes hydrological connectivity as water-mediated transfer of matter, energy and organisms within the hydrological cycle. Due to different spatial scales, scientific backgrounds and perspectives, there exist different sub-definitions of the concept of hydrological connectivity (e.g., see overviews by [11,12]). One of these is the perception of longitudinal connectivity. According to Duvert *et al.* [12], this stands for sediment behavior between upland and lowland compartments of a landscape and is as yet poorly understood.

Intensive monitoring is one way to face this challenge of understanding SSD. There are several studies presenting river catchment SSD monitoring networks at a high spatial and temporal resolution [1]. The tracing of suspended sediments along catchments [3] and the connectivity of SSD in streams to outside-stream compartments like hillslopes and groundwater is a focus of present-day research [12]. A distinct boundary between two spatial compartments is the interface between river catchments and lakes that act as sediment source areas and sediment sinks, respectively. Several studies have touched on this issue of connectivity between catchments and lakes, in particular by investigating reservoir siltation based on remote sensing [7] or source tracing techniques like isotope or magnetic characterization [13].

However, studies that directly link SSD in catchments and reservoirs through monitoring of the spatial and temporal distribution of suspended sediment in both compartments are scarce. Linking the two compartments functionally, Dearing [14] described lake sediments as records of past catchment behavior. Swierczynski *et al.* [15] presented a 1,600 year chronology of seasonally resolved flood events that was reconstructed from varved lake sediments. Czymzik *et al.* [16] stressed the importance of flood chronologies from lake sediments as a prolongation of instrumental flood time series. In order to bridge the spatial gap between instrumental catchment data and varved lake sediments, it is also very important to have a good understanding of stratification and mixing processes within the lake. This understanding of intra-lake processes is required to better decipher the past from lacustrine sediment cores [17]. Drifters, satellite remote sensing techniques, conservative tracers and acoustic sensors have the capability to measure flow inside lakes [18–20]. However, only acoustic current meters allow continuous *in-situ* monitoring of flow velocities. To gain a better understanding of SSD based on long-term and comprehensive observation data, monitoring systems must fulfill the following requirements:

- Suspended sediment concentrations must be measured continuously at a high spatial and temporal resolution to comply with the expected process dynamics. Given that flood events have a duration of hours to days, depending to the hydrological boundary conditions and catchment characteristics [21], sub-hourly resolution should be the goal.
- Limno-physical and meteorological boundary conditions acting as forcing parameters for SSD must be measured to resolve, e.g., the effect of mixing and stratification processes.
- Suspended sediment monitoring in the lake should be consistent with hydro-sedimentological monitoring stations within a catchment in terms of the parameters measured and its temporal resolution to capture the effects of flood-related water and sediment inflow from contributing streams.
- The system must be able to be deployed and operated at one or more fixed locations within the lake throughout the year.
- Accessibility, low maintenance effort and flexibility of the system's structure must be ensured.

Buoy-based monitoring systems are potentially a very valuable tool for monitoring sediment dynamics, mixing and stratification directly within water bodies, and thus for establishing the link between catchment processes and lake/reservoir SSD. Buoy systems have, for instance, been developed for studying water quality [22–26]. Alcântara *et al.* [26] applied the SIMA buoy system to understand turbidity behavior in the Amazonian floodplain and density currents in a Brazilian reservoir. Another example is the Chesapeake Bay Interpretive Buoy System [24], which is a sophisticated system that includes measurement of meteorological parameters, current measurements and point-measurement of water quality parameters and turbidity. The GLUCOS [25] observation system was applied in Lake Michigan to measure dynamics of spring stratification and water quality parameters. The Simpatico system developed by Garel *et al.* [23] used a YSI buoy system (YSI.com) deployed in a Portuguese estuary in combination with a turbidity measuring multi-parameter probe and two acoustic current sensors.

Existing buoy systems presented in the literature, however, do not fully satisfy the needs for exploring processes of SSD in lakes or estuaries as listed above. Systems introduced by e.g., Garel *et al.* [23] and Alcântara *et al.* [26] are equipped with single-point turbidity sensor systems and contain no or only single-sensor flow velocity measurements. The limitation of existing systems in particular is the low vertical resolution within the water column, the non-simultaneous measurement in different water depths and the problem of blanking by single-sensor flow velocity measurements. Blanking is the distance from transducers that is required before the sensor is able to obtain valid data. Minimum blanking distance for general purpose Acoustic Doppler Current Profilers is 20–25 cm [27].

In this study we present the design, deployment and initial datasets of a buoy system that meets the above challenges of a lake monitoring system. What sets the system apart from existing buoy systems mainly is: (a) the ability of simultaneous multi-level turbidity, temperature and electrical conductivity measurements; (b) highly resolved flow velocity measurement by two sensors, whereas one sensor is applied for surface and one for flow velocity of the whole water column, which guarantees maximum spatial coverage and resolution; (c) maximum maintainability due to easy accessibility of all sensor units.

## 2. Monitoring System

### 2.1. Design of the Buoy

**Figure 1.**
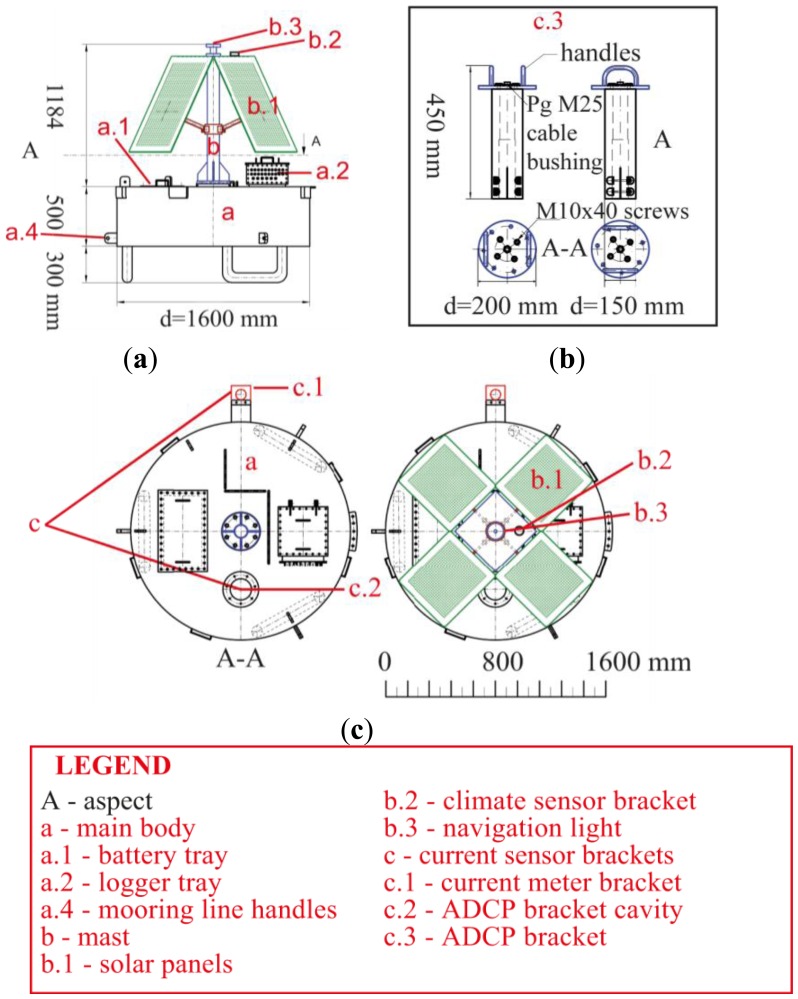
Technical drawing of the buoy. (**a**) The side view, (**b,c**) The top view of the main body and the entire buoy. The measurement chain is not illustrated. It is attached to the center of the bottom side of the main body.

The buoy design is focused on system flexibility in the sense of easy extension or modification of the sensor configuration and easy maintenance in terms of accessibility and frequency. The buoy consists of a main body (see Figure 1a), a mast (see Figure 1a) as carrier for the solar panels (see Figure 1c), the mounting for the meteorological sensor unit (see Figure 1c) and the navigation light (see Figure 1c). The body of the buoy serves as enclosure for the battery pack and the logger, as fixture for the measurement chain and as a lifting body. The buoyancy of the lifting body amounts to one cubic meter.

The cylindrical shape of the main body (MB) with its large top face area and comparatively small height was chosen to have enough interior space for technical equipment and easy accessibility for maintenance purposes, as well as low pitch and roll behavior and sufficient buoyancy in operation mode. Easy accessibility from a small boat was the main reason for the position of the two openings on the top face of the MB. As shown in Figure 1, the first tray (see Figure 1a) allows access to the batteries, and the second tray (see Figure 1a) to the data logger and communication equipment. The MB provides two places for acoustic current sensors. The integrated cylindrical cavity on the top side (see Figure 1c) next to the mast holds an Acoustic Doppler Current Profiler (ADCP). A special bracket was constructed (see Figure 1b) that assures easy accessibility and maintenance of the ADCP. To hold an Acoustic Doppler Current Meter (ADCM) in place, a clamp-like mounting device (see Figure 1c) exists at the lateral area of the cylindrical MB.

**Figure 2.**
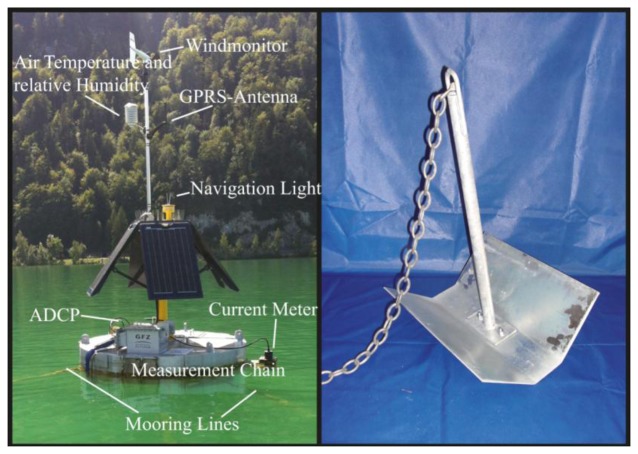
Buoy in operation and plate anchor as used for the mooring.

Tourism in the region of the deployment area was the main driver for defining the outer appearance of the buoy. It is a compromise between reduced perceptibility in contrast to the surrounding environment and a clear visibility of the buoy for shipping traffic (see Figure 2). Therefore, the MB is kept silver and the mast painted yellow. Buoy visibility at night and in poor weather conditions is assured by a programmable navigation light.

### 2.2. Energy Supply and Mooring

The energy supply system (ESS) of the buoy consists of a battery module, a solar charge controller and four solar panels. The battery module is made of four bridged 33 Ampere hour batteries. The bridged connection of the four batteries leads to a total energy storage capacity of 132 Amperes. The batteries are charged by four solar panels (Solara S160M136) with 45 Wp each. With the umbrella-like construction of the mounting plates at the mast (see Figure 1), the solar panels can be folded out up to an angle of 90 degrees. This makes them adjustable to different altitudes of the sun throughout the year to reach a maximum energy yield. Charging of batteries and preventing exhaustive discharge is managed by a Phocos PL20 solar charge controller. The technical setup of the ESS guarantees an efficient balance of charge and discharge of the batteries. Assuming low energy input due to poor weather conditions and a maximum power consumption of 13.8 Ampere hours per day (Ah/d) at 12 V, the system enables a minimum redundancy of 7 days. The logging unit has an energy load of 0.5 Ah/d, the sensors 11.4 Ah/d and the navigation light 1.9 Ah/d.

The mooring of every buoy is composed of three plate anchors made by Uwitec (see Figure 2). Plate anchors were chosen because of the very fine sediments in the deployment area (Lake Mondsee) with a mostly fine sandy to coarse silty texture [15]. Three mooring line handlers are evenly distributed around the MB of the buoy as can be seen in Figures 1 and 2. The number of tie points prevents a twisting around the vertical axis of the buoy. Based on previous experience with the mooring of drilling platforms, the length of the mooring lines is three times the water depth at the buoy position.

### 2.3. Sensor Equipment, Data Logging and Communication

The sensor equipment of the buoy consists of three main units (water quality, current and climate, see Table 1).

As an indirect measure of suspended sediment concentrations, turbidity is continuously and simultaneously recorded at nine different water depths to reach a high vertical resolution across the water column with a depth of about 23 m in the test deployment. Nine Forest Technology Systems, Inc. (FTS) (http://www.ftsenvironmental.com) nephelometric turbidity sensors were distributed along the measurement chain. Table 2 presents detailed information regarding the sensor distribution. The FTS-sensors have wipers as cleaning systems that prevent the optical face of the sensor from fouling by cleaning it every 15 min. According to the manufacturer, the DTS-12 uses true nephelometric geometry, which improves the signal-to-noise ratio by measuring the forward and backscattered light at 90° angle to the light beam. In order to guarantee extreme accuracy the built-in microprocessor takes 100 readings over 5 s and then computes and outputs turbidity values (accuracy and range see Table 3). An Uwitec Water Sampler (www.uwitec.at) is used to collect water samples at every depth in order to calibrate the turbidity sensors against suspended sediment concentrations from the water samples based on a linear regression [28]. Besides turbidity, the FTS DTS-12 sensors also measure water temperature. To assure a certain redundancy in water temperature measurements and to collect data on electrical conductivity as a proxy for total dissolved solids, and potentially for water density changes and suspended sediment concentrations, Campbell Scientific, Inc. (CS, http://www.campbellsci.com) CS547A-L electrical conductivity and temperature sensors were added to the measurement chain at similar depths as the turbidity sensors (Table 2). The measurement chain is fixed at the centre underneath the MB of the buoy. An anchor rope with a diameter of 10 mm is the backbone of the chain to which the single sensors are fixed. The top end of the chain is equipped with a leader for easy handling and recovery of the chain or of single sensors, and to the bottom end a sinker was fixed to keep the chain taut.

Retrieving information on flow conditions at a high spatial resolution is crucial for understanding the limno-physical boundary conditions, e.g., flow paths, currents, and thus the resulting sediment distribution inside the water body. Near-surface flow speed and direction are measured by a Nortek (http://www.nortek-as.com) Aquadopp Current Meter (ADCM) with a 2 MHz frequency and a 0.75 m measurement cell size in a water depth of 0.3 m (see Table 2 for detailed specifications), resulting in an integral signal from 0.3–1.05 m water depth. A Nortek Aquadopp Profiler (ADCP) is used to record these parameters in a profile from 1.5 m below lake level to the bottom of the lake with a cell size of 2 m. ADCM and ADCP follow the same principles of measurement based on three acoustic beams. The only difference is that the ADCP records an integral signal of flow velocity and flow direction for every depth increment, while the ADCM measures this signal for every single beam in one depth. An ADCP is used to determine current velocities by using the Doppler shift of a backscattered acoustic signal [29]. The choice of the acoustic frequency of the ADCP reflects a compromise between the desired vertical resolution and the water depth at the site. On the one hand, a higher operation frequency leads to smaller cell sizes and higher vertical resolution, and on the other hand, to a lower depth range of the ADCP. In the present configuration, the ADCP operates at a frequency of 400 kHz. This sensor offers a profiling range of 60 to 90 m and a cell size of 2–8 m. For the test deployment, a 2 m cell size was chosen. The frequency of 2 MHz of the ADCM is a default by the manufacturer.

To understand limno-physical processes such as mixing and stratification it is important to have information about the meteorological conditions close to the lake's surface [17]. Thus, meteorological sensors are part of the buoy system (Table 1). Wind speed and wind direction are recorded with a RM Young (http://www.youngusa.com) Wind Monitor 5103. Relative humidity and air temperature are recorded by an UMS-TempRH (http://www.ums-muc.de) (RFT-2) sensor. The climate sensors were mounted onto the mast at an elevation of 2 m above lake level for the TempRH sensor and 2.5 m for the wind monitor (Figure 2, Table 2).

Sensor data are logged by a Campbell Scientific (CS) CR1000-Logger. In connection with a CS AM16/32 Relay Multiplexer for measurement channel extension, a memory extension of 2 GB with a CS CFM100 CompactFlash Module and remote access with a CS GPRS-Modem kit, the logging unit is a powerful and easily extendible platform for the buoy system. Remote communication provides the possibility of data push from the buoy to a data server at the institute and remote control. The buoy pushes the recorded data in daily intervals to a data server.

### 2.4. Study Site and Test Deployment

**Figure 3.**
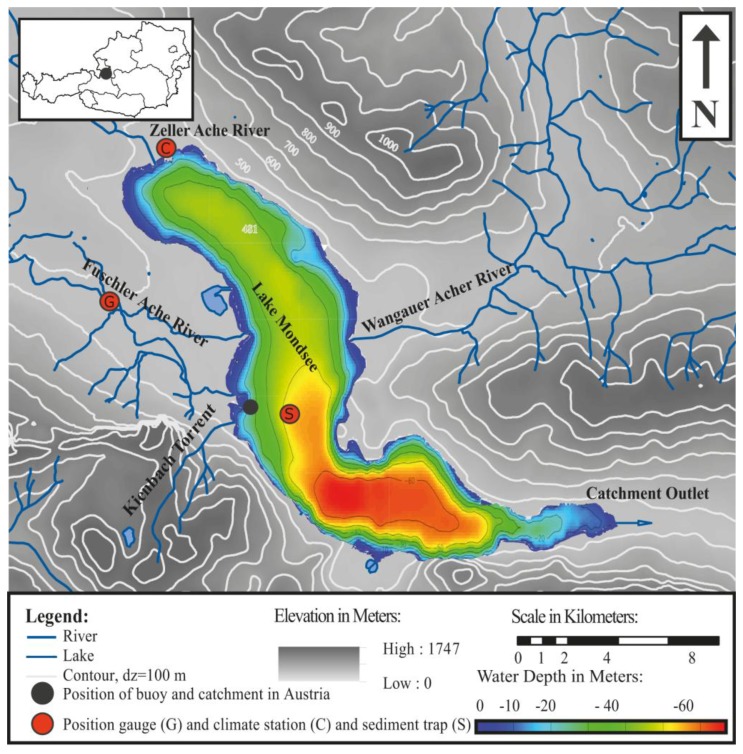
Map of Lake Mondsee and buoy position for test deployment. (C) Meteorological gauge operated by the ZAMG (Austrian Meteorological Survey). (G) Hydro-sedimentological gauge at the catchment outlet. (S) Position of the sequential sediment trap. The big black dot on the bathymetric lake map stands for position of the buoy during test deployment. The small black dot in the upper left corner shows the study site relative to the map of Austria.

The buoy was deployed on Lake Mondsee for test operation. The lake is located in the Austrian Pre-Alps, 40 km east of Salzburg within the federal state of Upper Austria (see Figure 3). The lake has a length of about 11 km, a maximum width of 1.5 km, a total surface area of about 14.2 km^2^, a mean water level elevation of 481 m above sea level and a maximum depth of 68 m.

Lake Mondsee is a dimictic lake [30]. Mixing covers the entire water column of the shallower parts, including the deployment position. Depending on weather conditions, in some years the lake is covered by ice in the winter season. This causes winter stratification and a dimictic regime, in contrast to mild winter seasons without ice cover and a mixing period from fall to spring without stratification during winter. The lake is defined by three major and several minor contributing catchments and one outlet (Figure 3), with a total catchment area of 247 km^2^. The northern part of Lake Mondsee is surrounded by an undulating pre-alpine landscape and the southern part by a steep alpine landscape. The geology of the catchment is dominated by a Flysch zone in the northern part and calcareous rocks in the southern part [15].

For a test application, the buoy was deployed close to and slightly south of the mouth of the Fuschler Ache River (Figure 3) into Lake Mondsee. Fuschler Ache River is the most important river draining into Lake Mondsee in terms of mean annual discharge. At the test position (47.824987 Decimal Degrees North, 13.370326 Decimal Degrees East) the lake has a depth of 23 m. As a reference system for the turbidity measurements, a Technicap PPS 4/3 (http://www.technicap.com) sediment trap was used. This trap was deployed in 2011 in a water depth of 55 m. It collects sediment samples sequentially in a temporal resolution of three days. The position of this trap relative to the buoy is shown in Figure 3.

## 3. Results and Discussion

During test operation of the buoy from August to November 2012, almost four months of data were recorded without interruption. Regarding the power management, battery voltage bridged poor weather conditions with low insulation and did not reach any critical level with a minimum of 12.63 V. In the following, the data collected during this period are presented and checked for plausibility.

### 3.1. Meteorological Data

**Figure 4.**
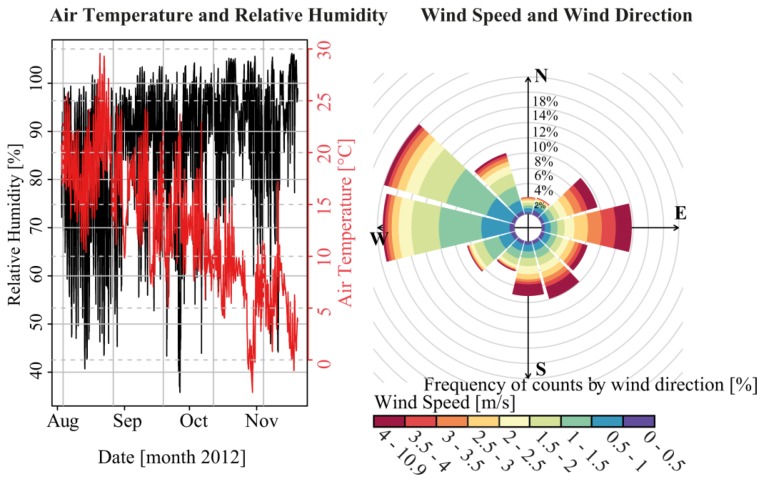
Measured air temperature and relative humidity (**left**) as well as wind speed in relation to wind direction (**right**).

Air temperature and relative humidity data show plausible ranges with clear diurnal cycles (Figure 4). Mean air temperature and relative humidity for the study period were 12.4 °C and 86.4%, respectively. These data are similar to a meteorological gauge (location shown as label G in Figure 3) operated by the Central Institute for Meteorology and Geodynamics of Austria (ZAMG) in the town of Mondsee at a distance of 3 km from the buoy. The long-term data of the ZAMG station for August–November (1964–2000) were 11.3 °C and 80.2%. Air temperature during the deployment period was one degree Celsius warmer than the long term average from 1964–2000. Wind velocity recorded by the buoy are plotted in a wind rose diagram (Figure 4). Westerly to northwesterly, followed by northeasterly to southeasterly wind directions clearly dominated during the operation period. This is in line with the topographic situation in the surroundings of the buoy, with high mountain ranges blocking direct access from other directions (Figure 3). The two main wind directions were characterized by marked differences in the distribution of wind speed. For northwesterly directions, wind speeds were mainly in the range of 0.5 to 2 m/s and only a minor section had wind speeds greater than 2 m/s. The opposite is the case for northeasterly to easterly winds. These directions were dominated by wind speeds greater than 2.5 m/s up to 11 m/s. Due to the complex topography surrounding Lake Mondsee and the resulting asymmetric wind fields of such valleys [31], it is difficult to check the plausibility of the recorded wind dynamics with other observations in the vicinity of the buoy. The long-term average wind speed (1964–2000) of 1.6 m/s for the months of August to November at the meteorological station of Mondsee lies within the same order of magnitude as the mean wind speed of 2 m/s measured at the buoy's location.

### 3.2. Water Quality Data

**Figure 5.**
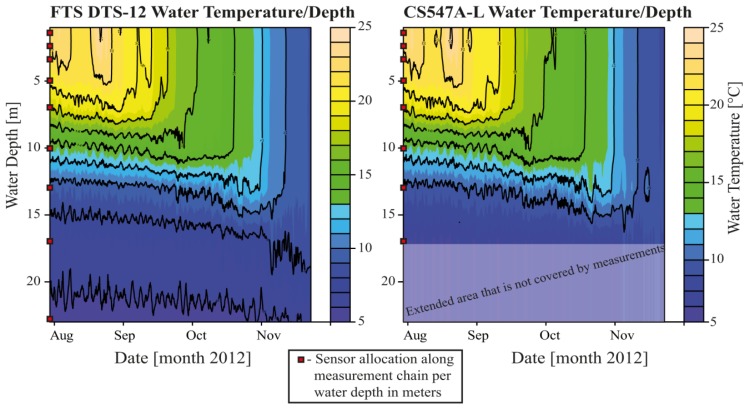
Measured water temperature by depth as a contour plot recorded by two different sensors. The FTS DTS-12 on the left and the CS547A-L on the right. Sensor allocation by water depth is illustrated by the red squares.

Water quality data include time series of water temperature, turbidity and electrical conductivity (Table 1). Figure 5 shows recorded water temperature as a function of depth and time for the two sensor types. The overall dynamics are very similar for both sensor types. Periods of stratification and mixing of the water column at the buoy location can clearly be identified. Summer stratification lasted until the end of October.

The warmer upper layer, the Epilimnion with temperatures ranging from 25 to 15 °C (see Figure 5) and low vertical temperature gradients, reached a depth of about 6 m in August and September. The Metalimnion with steep temperature gradients extended to a depth of about 13 m. The Hypolimnion was represented by water temperatures lower than 11 °C (see blue areas in Figure 5).

Temperature differences between the different layers started to diminish by the middle of September onward, until stratification broke down at the end of October and total mixing occurred. The water temperature data collected at the buoy location show the typical temporal dynamics of a holomictic or dimictic pre-alpine deep water lake with a period of stratification and a period of total mixing of the water column. Similar temperature ranges and stratification or mixing behavior of Lake Mondsee and other pre-alpine lakes have been shown in several studies [17,32]. This confirms the plausibility of the recorded water temperature data of the new buoy system.

**Figure 6.**
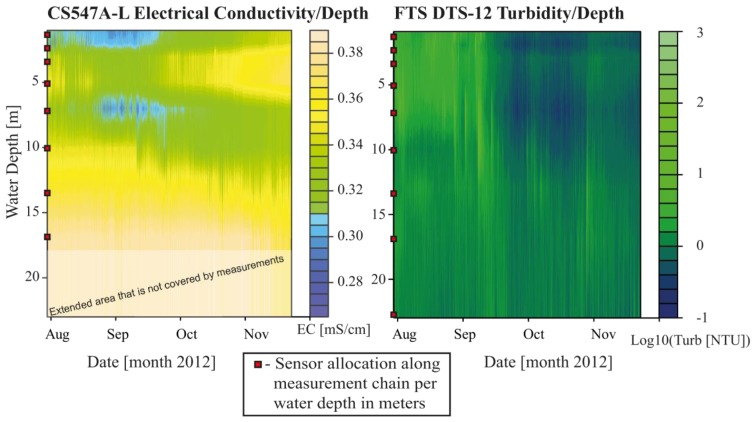
Electrical conductivity (CS547A-L, **left plot**) and turbidity (FTS DTS-12, **right plot**) displayed in contour plots at the buoy location. Sensor allocation by water depth is illustrated by the red squares. Turbidity is presented with a logarithmic scale.

Besides temperature, electrical conductivity (EC) and turbidity were recorded at different depths (Table 2). The deeper part, roughly comprising the Metalimnion and Hypolimnion ranging from about 10 to 23 m water depth, had constantly higher EC over time (>0.34 mS/cm) than the upper part of the profile (Figure 6). Furthermore, the upper part showed a larger variability of EC (0.26–0.38 mS/cm) within the observation period. Similar to temperature, the EC values tended towards homogenization over water depth in November, coinciding with the mixing of the water body. Thies *et al.* [33] presented a quite comparable EC range (0.015–0.5 mS/cm) for the alpine Lake Rasass in Austria.

Turbidity is the main parameter of the buoy system for continuously and indirectly measuring suspended sediment concentrations (SSC). Figure 6 shows the recorded turbidity interpolated over depth and time at the buoy location. Red rectangles represent the sensor positions over depth. Overall, there was very low temporal variability of turbidity values for most depths (see Table 3). Statistical parameters shown in Table 3 were derived from the turbidity time series of the entire test deployment period. Maximum values reflect the system's ability of displaying turbidity peaks during flood events. Low mean values with no increasing trend indicate the efficiency of the wiper-based cleaning system of the sensors, which avoided sensor fouling. Overall, this underlines the operational reliability of the turbidity sensors in the buoy system. Significant peaks in turbidity were only recorded for the sensors allocated at depths of 1 m, 2 m and 3 m during August and September. Maximum values and standard deviations of turbidity time series indicated decreasing dynamics of turbidity with depth (Table 3). At first glance, a maximum-recorded value of 618 NTU seems to be low, but considering the fact that it is more than one third of the sensor's total measurement range (see Table 2) and the diluting effect, which originates when confluent stream water mixes with lake water, the recorded values are reasonable. Notably, the observed turbidity events were mostly confined to the top 3 to 5 m (Figure 6). After event-related higher turbidity, the signal fell back to values close to zero. To further evaluate the observed turbidity data in the lake, the monitoring data of a hydro-sedimentological gauging station (see label C presented in Figure 3) at the outlet of the Fuschler Ache catchment into the lake were considered. In this way, one may distinguish between lake-internal factors causing high turbidity and flood-induced suspended sediment loads from the catchment. Sensor fouling, algae growth and backscattered light, among other factors, may influence observed turbidity with nephelometric turbidimeters in lakes and reservoirs [34–36]. Other processes that lead to high turbidities of lakes are the wind-induced re-suspension of sediment deposits [37] and turbidity currents [38]. Comparable to the buoy, the river gauge is also equipped with a FTS DTS-12 turbidity sensor and also with an automatically pumping water sampler and with water level sensors. The identical technical setup and type of turbidity sensors provide a valuable reference system for suspended sediment measurements at the buoy location, both in terms of the mere occurrence of suspended sediment pulses to the lake and in terms of their concentration.

**Figure 7.**
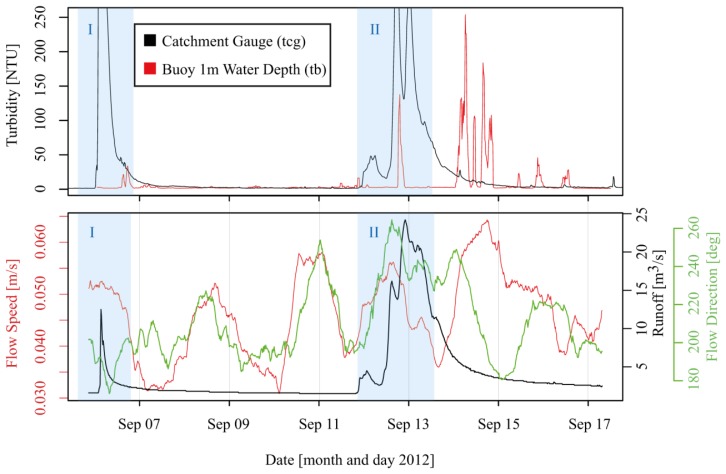
Comparison of turbidity in the Fuschler Ache river at the most downstream gauge (tcg) and of turbidity at the buoy location (tb) at 1 m water depth (**upper graph**). The position of the catchment gauge is represented by label C in Figure 3. River runoff, flow direction and surface flow speed at the buoy location (**lower graph**). I and II represent the two reference runoff events. Graphs shown in the upper plot are scaled with one y-axis. In the lower plot, each graph corresponds to the y-axis of the same color.

For two runoff events, the lower part of Figure 7 shows hourly time series of turbidity and water flow averaged from monitoring data with 15-min resolution. Turbidity from the buoy (upper graph) was filtered to an hourly resolution. The comparison shows that turbidity peaks at the catchment gauge (Figure 7 tcg and Figure 3C) were accompanied by turbidity events at the buoy location (Figure 7 tb). However, the timing and magnitude of the turbidity increase at tb were different for the two events. For the first event that occured between the 5th and 6th of September (event I in Figure 7), a pronounced but short turbidity peak was observed at the buoy during the first part of the flood wave. Maximum correlation with r^2^ = 0.64 (n = 192, n-number of 15-min time steps) between the turbidity time series at the catchment gauge and at the buoy was achieved for a lag time of almost 28 h. For the event occurring between the 12th and 15th of September (event II in Figure 7), the response of the buoy to a slight increase in turbidity was about 14 h after the first major peak of the river gauge that is approximately 2 km upstream in the Fuschler Ache river relative to the buoy location. For this time lag, the highest and most significant correlation of r^2^ = 0.49 (n = 576, n-number of 15-min time steps) between the turbidity pulse in the catchment and the recorded response at the buoy was calculated. Whether these late signals were still related to suspended sediment input from the Fuschler Ache river by the runoff event cannot be unambiguously resolved based on the present preliminary data analysis. Sediment that entered the lake from the two other contributing catchments (Wangauer Ache, Zeller Ache—see Figure 3) or circular flow patterns within Lake Mondsee [39] that distributed the sediments in different ways at different limno-physical boundary conditions and may have caused a delay between sediment entering the lake and its appearance at the particular buoy location. Alternatively, growth in algae populations may cause sudden rises in turbidity [34]. Dokulil and Skolaut [40] reported peaks in phytoplankton populations in Lake Mondsee during the end of August and September. Furthermore, they report during this period the maximum total biovolume consisting of different phytoplankton species in the topmost 5 m of the water column. This circumstance may explain the high turbidity values recorded during times when no flood event took place in the contributing catchment.

**Figure 8.**
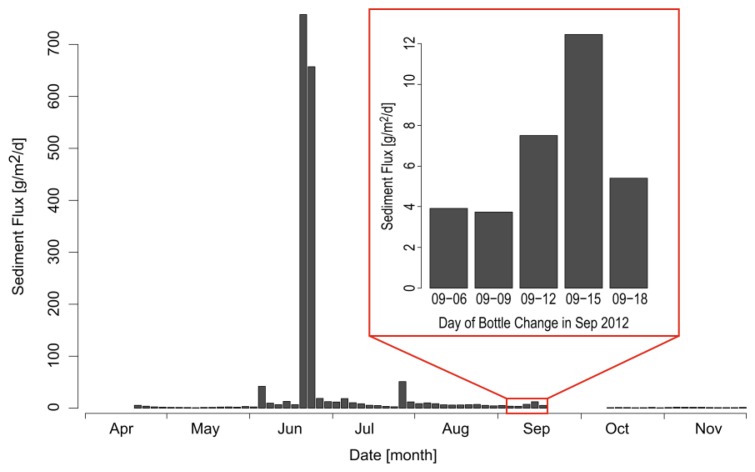
Sedimentation rates from a Technicap PPS4/3 sediment trap working sequentially with a temporal resolution of three days. Sedimentation rates are shown from April to November 2012. The red frame highlights the sedimentation rated during the flood event.

However, in order to strengthen the hypothesis that recorded turbidity peaks at the buoys are related to suspended sediment transported during the reference events (Figure 7: I and II), sediment trap data were additionally taken into account. Figure 8 shows sediment flux trapped by the sequential sediment trap from April to November 2012. The time period of the flood events discussed before is highlighted in Figure 8. The number of samples in April and between the end of September and the beginning of October is lower than in all other months (see Figure 8). In April this was due to ice coverage of Lake Mondsee and in the period between September and October the vessel that was used for surveys was not available, so that the sampling bottles could not be changed.

Highest sedimentation rates were recorded for the period 13th to 15th of September, in line with the timing of the major turbidity peaks at the buoy location. Even several days after the runoff event (16th to 18th of September), a higher sedimentation rate was recorded at the trap, which corresponds to several late and smaller turbidity peaks at the buoy. Thus, measured turbidity at the buoy location can be expected to be a sediment signal and not related to algae growth. The selected events show that sediment pulses entering the lake produce diverse responses at the buoy location in the lake. This is an evidence of complex flow patterns in Lake Mondsee.

A rating curve as a calibration function for transferring turbidity to SSC has not yet been established. For the test deployment period, only samples covering the low range of turbidity values were able to be taken because of the low number of flood events. This problem is not general, but related to the particularities of the test period which was a period of overall low sediment input into Lake Mondsee compared to other parts of the year 2012, as seen from the sediment trap data (Figure 8).

### 3.3. Current Data

**Figure 9.**
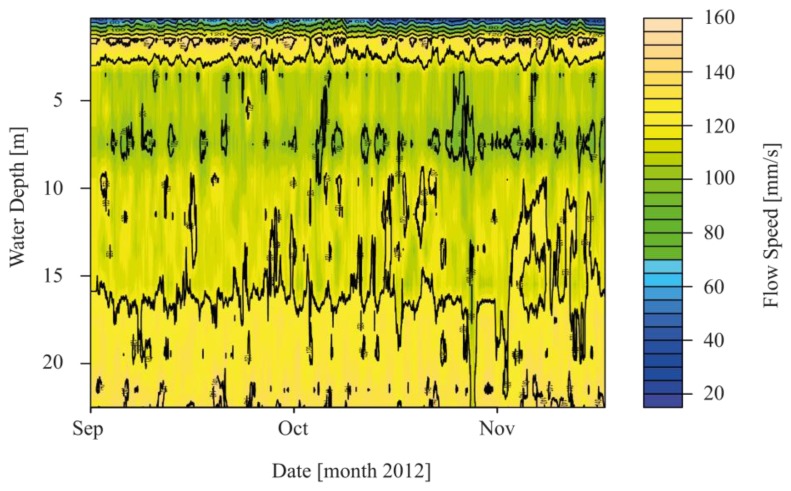
Interpolated flow speed (fs) from ADCP and current meter data at the buoy location.

Flow speed (fs) and flow direction (fd) were recorded from 1st of September–18th of November without failure of either the ADCP or the ADCM current meter. Data for the first weeks of deployment in August 2012 are incomplete for both sensors because of problems with the cable connection from the sensors to the logger. Figure 9 shows the interpolated flow profile over depth and time recorded by the current meter at 0.3 m water depth and the ADCP from 1.5 to 23 m water depth. Plotted flow speed represents the horizontal flow components of each measurement cell. Current data at an hourly resolution were aggregated to 24-hour resolution in order to enhance the signal-to-noise ratio.

**Figure 10.**
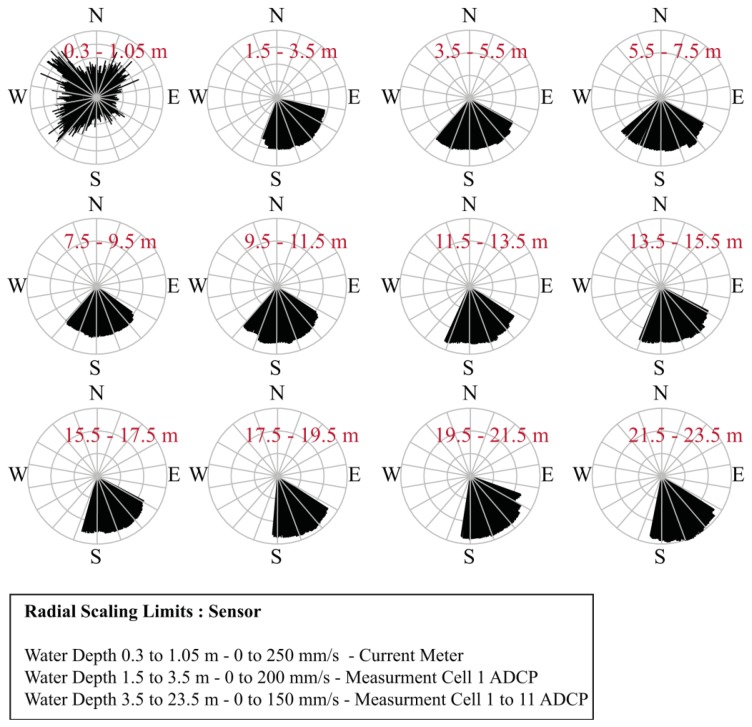
Flow directions over all depths per cardinal direction recorded by current meter and acoustic doppler current profiler. The ranges listed in red represent the measurement water depths. Only the range from 0.3 to 1.05 m was recorded by the acoustic doppler current meter (ADCM). The remaining data were recorded by the acoustic doppler current profiler (ADCP). Data shown covers the period from 1st of September to 18th of November.

Very dynamic patterns of fs can be seen in the topmost 2–3 m of the water column. Particularly the flow speed in the topmost 1 m and in the first two cells of the ADCP shows a large variability over the entire measurement period. Surface flow speed measured by the current meter was 44 mm/s on average and 243 mm/s maximum for the study period. For ADCP, the mean and maximum were 142 mm/s and 211 mm/s for the first cell (1.5–3.5 m depth) and 109 mm/s and 171 mm/s for the second cell (3.5–5.5 m depth). Standard deviations (sd) of the time series of the current meter fv, sd = 30 mm/s, cell 1 of the ADCP fs, sd = 54 mm/s and cell 2 fs, sd = 37 mm/s point to the fact that the variability around the mean in the recorded values is the highest at a water depth of 1.5 to 3.5 m, followed by fs recorded at 3.5–5.5 m water depth and the topmost values recorded by the current meter. In deeper parts from 5.5 m to approximately 17 m a decrease in flow speed was observed (Figure 9). Below 17 m fs increases again, with the highest fs at a depth of 22 m with a mean of 131 mm/s and a maximum value of 207 mm/s. Remarkably, flow speed at 0.31–1 m water depth is lower than in adjacent deeper layers. This effect was also observed by Boehrer *et al.* [41] in a study of the vertical structure of currents in western Lake Constance. In Figure 10, flow velocities for the different depths are shown and can be compared with the distribution of wind directions (Figure 4).

Flow directions in the surface layer recorded by the current meter were considerably different than those collected by the ADCP in the deeper layers. A comparison of the current meter data (Figure 10) with the wind data (Figure 4) reveals that the shallower flow regime dominated by westerly flow directions (0–1.05 m water depth) compares well with the predominance of westerly wind directions. In contrast, the deeper flow regime dominated by southeasterly flow directions (1.5–23 m water depth) can be clearly distinguished. An earlier study of the flow regime in Lake Mondsee by Ferencik [40] confirms these observations. Based on intensive flow measurement campaigns at 5 m and 20 m water depths at Lake Mondsee, Ferencik [40] showed that a clockwise rotating eddy formed over the largest part of the year as a consequence of the local and larger-scale wind regime, the special topography of the surrounding landscape and the lake basin, as well as the spatial distribution of the contributing streams around the lake (see Figure 3). This leads to a main flow direction from south to north in the area of the outlet of the Fuschler Ache River into Lake Mondsee, *i.e.*, the buoy location. As described in this study, only consistent southerly winds cause an inversion of this eddy. All other local wind directions speed up to 10 m/s that are described as smooth and temperate winds for Lake Mondsee cause clockwise circulation [40] and thus fit to the measured data retrieved by the buoy.

However, in view of the campaign-like data retrieval with drifters in the study of Ferencik [40], no final conclusions about seasonality or stationarity of the flow regime in Lake Mondsee could be made. Further investigations with longer and spatially distributed buoy deployment are expected to give more insight into the flow dynamics in Lake Mondsee. Regarding the turbidity data recorded at the buoy (see above), this complex flow regime could be one reason for turbidity peaks that are not related to flood-prone sediments from the Fuschler Ache river catchment. Concerning the range of recorded flow speed, the two studies of pre-alpine lakes prove the measured ranges of up to 243 mm/s near the water surface or even 207 mm/s at a water depth of 22 m. Ferencik [40] reports speeds around 200 mm/s even in greater water depths for Lake Mondsee, and Boehrer *et al.* [41] report flow speeds of the same order of magnitude for Lake Constance.

## 4. Conclusions

In this study, we present the design of a novel buoy system to monitor suspended sediment concentrations and related limno-physical and meteorological variables at high spatial and temporal resolution in lakes or reservoirs. High temporal resolution was achieved by recording all sensors at 15 min intervals. At the same time, high spatial resolution in resolving the vertical profile of the water column is achieved by means of parallel measurements at several water depths. For the test deployment period of several months, the buoy system proved to be a valuable tool for monitoring lake dynamics, as all measurement units, power supply and data transfer functioned without failure and with data ranges that were in the order of magnitude of similar observations in other studies. Furthermore, the buoy was not shifted or tilted and maintained operation even during strong wind or current conditions during the deployment period. This confirms the functionality of the mooring concept and the stability of the buoy design itself, including energy management, sensor equipment and logger system.

Recorded water temperature data clearly showed seasonal stratification and mixing of the water body in Lake Mondsee within the test deployment period. Turbidity data point out that there are certain limitations in straight-forward measuring of suspended sediment concentrations with the indirect method of nephelometric turbidimetry. Unfortunately it was not possible to collect enough samples to establish a rating curve to derive SSC from turbidity. This was mostly due to the small number of flood events during the deployment period and, thus, for a limited range of turbidity and SSC values. During upcoming flood events, particularly during the main flood season in spring and summer, sampling campaigns will be conducted in order to obtain lake water samples for each turbidity sensor location. These samples will be analyzed according to their bulk sediment mass and grain size distribution with the objective to establish event-specific and sensor-specific relations between turbidity and suspended sediment concentration. Another strategy pursued in future is to obtain continuous information on suspended sediment concentration by using the ADCP measurements. Previous studies have gone this way by conducting ADCP measurement campaigns in order to investigate processes of suspended sediment transport within lakes (e.g., [29,42].) But in contrast to these studies, which only deliver temporal snapshots, the presented buoy system should be able to collect continuous sediment data derived from an ADCP. A dual approach that derives suspended sediment concentrations from both turbidity and ADCP measurements may lead to more robust estimates. Furthermore, reference measurements and further investigation of algae migration and populations is necessary to clearly distinguish *in-situ* lake turbidity from flood-related sediment pulses or other turbidity-causing boundary conditions. During the test deployment period, a hydro-sedimentological river gauge as reference for identifying flood signals from the catchment was used. Despite the low number of events, it was possible to demonstrate on the basis of the two biggest flood events that floods and related suspended sediments were transferred from the catchment to the buoy location.

Analysis of flow patterns at the buoy location based on acoustic current measurements revealed two flow regimes in this part of Lake Mondsee. The first regime is limited to the topmost 1 m of the water column and is strongly influenced by local winds. The second regime ranges from approximately 1.5 m to 23 m water depth. It runs opposite the surface currents and the overall flow direction of the lake from north to south (lake inflows in northern and central parts, lake outflow in the very south). In line with an earlier study, this finding points to larger-scale circular currents within Lake Mondsee. To test this hypothesis and to evaluate the currents' effect on suspended sediment dynamics within the lake, further investigations are needed. The findings on flow patterns in the lake might be of great importance to other fields such as paleo-limnology, where paleo-chronologies of flood events based on laminated lake sediments are developed [15,16]. Transport mechanisms for sediments within the lake as a function of, e.g., stratification and circular current dynamics may need to be explicitly taken into account for these studies. Only in this way may it be possible to relate flood-related sediment input from one or several catchments unambiguously to the presence or absence of laminations in the sediments at the core location and to set up valid geo-chronologies. Investigations in this direction call for the extension of the buoy monitoring system to several locations across the lake and to the detailed analysis of the role of vertical water movement and solute transport *versus* the lateral processes. To this end, the combination of diverse monitoring data with modelling of lake dynamics is a further objective.

In summary, it can be stated that the buoy system presented here is a very valuable tool for continuously measuring suspended sediment dynamics at a high spatial and temporal resolution within lakes. The unique combination of instruments that resolve physical parameters related to sediment transport processes continuously in time and with a high spatial resolution along the water column may be one step forward in understanding depositional processes within lakes. This could be a way to test existing hypotheses on depositional processes within lakes [43,44] as well as the seasonal behavior of these processes with the aim to understand the information content of lacustrine sediments with regard to flood frequencies and magnitudes. For hydrological sciences, this could be a way to achieve more reliable long-term data on flood chronologies through understanding the *in-situ* processes in lakes.

## Figures and Tables

**Table 1.** Measured parameters of the buoy system.

**Water Quality** **Parameter [Unit]**	**Current** **Parameter [Unit]**	**Meteorology** **Parameter [Unit]**
Turbidity [NTU] Water Temperature [°C] Electrical Conductivity [mS/m]	Flow Speed [mm/s] Flow Direction [deg/10]	Relative Humidity [%] Air Temperature [°C] Wind Speed [m/s] Wind Direction [deg]

**Table 2.** Allocation and technical overview of sensors attached to buoy during test operation. Logging interval for all sensors is ∂t = 15 min.

**Type of Sensor**	**Parameter*[Range/Accuracy]***	**Type of Assembly**	**Allocation Relative to Water Body**
FTS DTS-12 (Nephelometric turbidity sensor)	Turbidity *[0–1600 NTU (nominal*)/±*2*% *of reading (0*–*399 NTU)*, ±*4*% *of reading (400*–*1,600 NTU)]* and water temperature*[0 °C*–*40 °C/*±*0.2 °C]*	At different water depths along measurement chain	Water depths along measurement chain in meters: 1, 2, 3, 5, 7, 10, 13, 17, 23
CS547 A-L	Electrical conductivity [approx. 0.005–7.0 mScm^−1^/±*5*% *of reading 0.44 to 7.0* mScm^−1^*and* ±*10*% *of reading 0.005*–*0.44* mScm^−1^ *for standard solutions*] and water temperature *[0 °C*–*50 °C/*±*0.4 °C]*	At different water depths along measurement chain	Water depths along measurement chain in meters: 1, 2, 3, 7, 10, 13, 17
Nortek Aquadopp Current Meter (acoustic Doppler current meter at 2 MHz)	Flow speed *[*±*5 ms*^−^*^1^/1*% *of measured value* ±*0.5 cms*^−^*^1^]*and flow direction *[Azimuth: 360°/2°/resp. 0.1° for tilt* <*20°]*	Attached to MB of buoy	0.3 m water depth with a measurement cell size of 0.75 m
Nortek Aquadopp Profiler (acoustic Doppler current profiler at 400 kHz)	Flow speed *[*±*10 m/s/1*% *of measured value* ±*0.5 cm/s]* and flow direction *[Azimuth: 360°/2°/resp. 0.1° for tilt* <*20°]*	Attached to MB of buoy	0.5 m water depth with 1.0 m blanking distance and with measurement cell size of 2 m; resulting measurement range is from 1.5 to 21.5 m
RM Young Wind Monitor	Wind *speed [0*–*100 ms*^−^*^1^/[*± *0.3 ms*^−^*^1^ or 1*% *of measured value]* and wind direction *[Azimuth: 360° mechanical, 355° electrical (5° open)/*±*3°]*	Attached to mast of buoy	2.5 m above water surface
UMS TempRH	Air temperature *[*−*40 °C*–*80 °C]* and relative humidity *[0%*–*100*% *rH/0.01 kPa]*	Attached to mast of buoy	2.0 m above water surface

**Table 3.** Primary statistical characteristics of turbidity data collected from sensors in all monitoring depths for the entire observation period (n = 12,201).

**Sensor Allocation**	**MEAN [NTU]**	**MAX [NTU]**	**STD [NTU]**
1 m	2.97	448.08	13.85
2 m	2.59	618.43	14.02
3 m	2.29	481.16	9.84
5 m	1.73	121.21	1.98
7 m	1.70	45.75	2.27
10 m	1.176	35.700	1.29
13 m	1.333	67.430	0.87
17 m	1.21	13.08	0.42
23 m	1.078	22.450	0.42
